# Phosphonic Acid Coupling Agent Modification of HAP Nanoparticles: Interfacial Effects in PLLA/HAP Bone Scaffold

**DOI:** 10.3390/polym12010199

**Published:** 2020-01-13

**Authors:** Cijun Shuai, Li Yu, Wenjing Yang, Shuping Peng, Yancheng Zhong, Pei Feng

**Affiliations:** 1 State Key Laboratory of High Performance Complex Manufacturing, College of Mechanical and Electrical Engineering, Central South University, Changsha 410083, China; 2 Institute of Bioadditive Manufacturing, Jiangxi University of Science and Technology, Nanchang 330013, China; 3 Cancer Research Institute, School of Basic Medical Sciences, Central South University, Changsha 410013, China

**Keywords:** interfacial bonding, coupling agent, HAP, scaffold

## Abstract

In order to improve the interfacial bonding between hydroxyapatite (HAP) and poly-l-lactic acid (PLLA), 2-Carboxyethylphosphonic acid (CEPA), a phosphonic acid coupling agent, was introduced to modify HAP nanoparticles. After this. the PLLA scaffold containing CEPA-modified HAP (C-HAP) was fabricated by selective laser sintering (frittage). The specific mechanism of interfacial bonding was that the PO_3_^2−^ of CEPA formed an electrovalent bond with the Ca^2+^ of HAP on one hand, and on the other hand, the –COOH of CEPA formed an ester bond with the –OH of PLLA via an esterification reaction. The results showed that C-HAP was homogeneously dispersed in the PLLA matrix and that it exhibited interconnected morphology pulled out from the PLLA matrix due to the enhanced interfacial bonding. As a result, the tensile strength and modulus of the scaffold with 20% C-HAP increased by 1.40 and 2.79 times compared to that of the scaffold with HAP, respectively. In addition, the scaffold could attract Ca^2+^ in simulated body fluid (SBF) solution by the phosphonic acid group to induce apatite layer formation and also release Ca^2+^ and PO_4_^3−^ by degradation to facilitate cell attachment, growth and proliferation.

## 1. Introduction

Recently, biopolymers and bioceramics composite materials have been widely applied to bone tissue engineering [1], which shows some features of natural bone in the main composition. As a result, the composite materials were designed to mimic the microstructure of cancellous and provide an ideal environment for bone regeneration. Poly-l-lactic acid (PLLA)/hydroxyapatite (HAP) becomes one of the representative composite materials, as they combine the bone-binding capabilities of HAP and the biocompatibility of PLLA [2,3]. Besides, incorporating HAP into the PLLA matrix would improve cell proliferation and viability and neutralize the acidic degradation products released from PLLA [4,5]. However, the weak interfacial bonding between HAP and PLLA is an obvious problem, which would bring about a low-stress failure at the interface, resulting in the deterioration of mechanical properties [6,7]. 

Coupling agents have been widely used to improve the interfacial bonding between biopolymers and bioceramics. For example, Khosravi et al. [8] modified montmorillonite (MMT) with coupling agent 3-glycidoxypropyltrimethoxysilane for good adhesion with PLA and reported that the –OH of 3-glycidoxypropyltrimethoxysilane reacted with the –OH of the montmorillonite surface forming a covalent bond. Rooj et al. [9] modified halloysite nanotube (HNT) by coupling agent bis (triethoxysilylpropyl)-tetrasulphide to improve the interfacial adhesion between HNTs and natural rubber, and results indicated that the –Eto of bis (triethoxysilylpropyl)-tetrasulphide had been connected covalently with the –OH of HNTs. In their studies, the types of coupling agent were always fixed while applying to specific bioceramics. Therefore, it is fair to say that for a specific ceramic, choosing an appropriate coupling agent with specific active functional groups to modify is really needed. 

Considering that HAP is a bioceramic with exchangeable phosphate ions, it may be accessible to introduce a phosphonic acid coupling agent to modify it due to their homology. Some research suggested that phosphonic groups have a high affinity to calcium phosphate-based biomaterials [10]. For example, Varma et al. [11] verified that phosphorylated chitosan could easily adhere to calcium phosphate. Shinzato et al. [12] reported that the phosphoric ester monomer had strong adhesion to bioactive glass. 2-Carboxyethylphosphonic acid (CEPA) was a phosphonic acid coupling agent containing carboxyl (–COOH), and the –COOH of CEPA could react with functional polymers having hydroxyl or amino groups, such as PLLA and Polyetherimide [13]. Previous study indicated that the –COOH of the material could occasion an esterification reaction with the –OH of the functionalized polymers [14]. Thus, CEPA might be considered as an appropriate choice for the surface modification of HAP to enhance interfacial bonding between HAP and PLLA. 

In this study, the CEPA coupling agent was introduced as a cross-linking bridge to improve the weak interfacial interaction between PLLA and HAP, and the PLLA/C-HAP scaffold was fabricated by selective laser sintering (SLS). This SLS is the most suitable 3D printing technique, which can fabricate the interconnected porous structure and personalized external shapes of the bone scaffold [15]. The microstructures of C-HAP particles and scaffolds were characterized by Fourier transmission infrared spectroscopy (FTIR), X-ray diffraction (XRD) and scanning electron microscopy (SEM). The wettability and thermal stability of the scaffolds were evaluated by water contact analysis (WCA) and thermogravimetry analysis (TGA). The mechanical tensile test was carried out to assess the effect of C-HAP particles on the mechanical properties of the scaffolds. In addition, the bioactivity of the scaffolds was assessed when it was incubated in simulated body fluid (SBF) solution, and the cytocompatibility was investigated by culturing MG-63 cells.

## 2. Materials and Methods 

### 2.1. Material and Scaffold Preparation

Poly-l-lactic acid (PLLA) (reagent grade > 99%) was purchased from Shenzhen Polymtek Biomaterial Co., Ltd. (Shenzhen, China). Nano hydroxyapatite (HAP) with a mean particle size of 100 nm was provided by Chengdu Organic Chemicals Co., Ltd. (Chengdu, China). In addition, 2-carboxyethylphosphonic acid (CEPA) was supplied by Nakhon Ratchasima, Thailand. The surface modification of HAP with CEPA was performed by a high-intensity ultrasonic probe, which was operated at 20 kHz with 100 W power. Briefly, the mixture of 5 g HAP and 1.54 g CEPA (1:1 molar ratio) was dispersed in an aqueous medium with a pH of 4–5 and sonicated for 4.5 h at room temperature. Subsequently, the product was filtered through a centrifugal filtration process. Finally, the modified HAP (C-HAP) powder was obtained after washing with deionized water and drying in a vacuum oven at 80 °C for 12 h. 

Preparation of HAP/PLLA or C-HAP/PLLA composite powder and scaffolds was carried out mainly through the following procedures: (a) certain amounts of PLLA, HAP and C-HAP powder were separately weighted according to the composition design (Table 1), and then the composite powder was respectively dispersed in ethanol; (b) the obtained solutions were ultrasonically dispersed for 45 min and magnetically stirred for 1 h, respectively; (c) finally, the uniformly dispersed solutions were filtered and dried to obtain composite powder for scaffold fabrication. 

**Figure 1:**
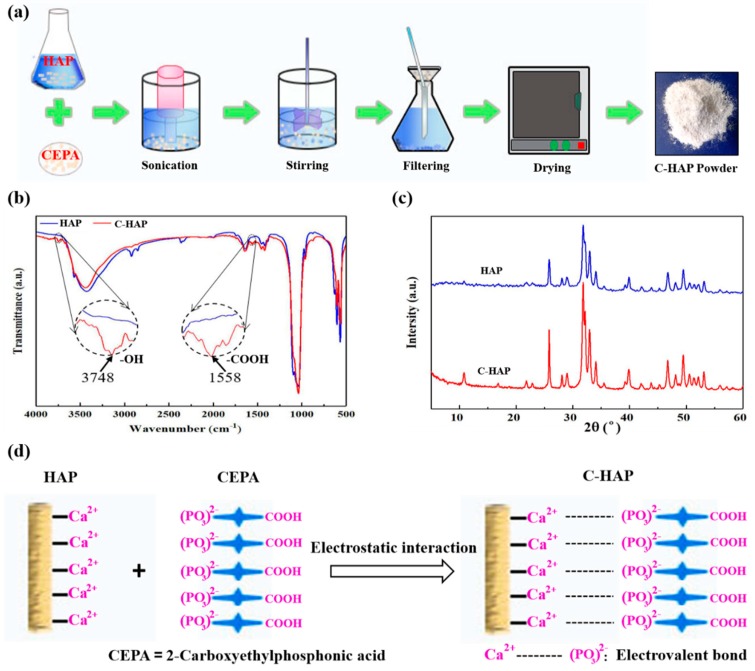
(**a**) The fabrication process of 2-carboxyethylphosphonic acid-modified hydroxyapatite (C-HAP) was powder, (**b**) Fourier transform infrared spectroscopy (FTIR) spectra and (**c**) X-ray diffractometry (XRD) pattern of C-HAP and hydroxyapatite (HAP), (**d**) Schematic of the possible interactions between HAP and C-HAP.

The selective laser sintering (SLS) system is composed of a CO_2_ laser, a three dimensional (3D) galvanometer scanner, and a computer-control system that was used to sinter thin layers of composite powder, forming 3D porous scaffolds. As shown in Figure 1, during SLS fabrication, the laser beam was selectively scanned over the powder surface according to the slicing data of the designed 3D scaffold models. In detail: Firstly, the computer-aided design (CAD) model of the sample was designed on the computer and converted into a stereolithography (STL) file. Then, the STL file was sliced to generate the laser processing path. Under the control of the computer, laser beam selectively scanned over the powder surface with the set scanning speed, laser power, scanning path, and so on. The powder at the laser scanning area absorbed energy and then was sintered to form a dense layer of a certain thickness, while the powder in the unirradiated area remained loose. After the slicing path processing of each layer was completed, the processing platform would decrease the height according to a thickness of the sintered layer, and the sintering of the next layer would start after the completion of the powder laying again. A new sintered layer would bond with the previous layer and repeat until all processes were completed. After all of the scanning steps, the unsintered powder around the specimen was removed to obtain the parts.

### 2.2. Properties Characterization

The phase analysis of the HAP powder, C-HAP powder and scaffold samples was done by X-ray diffractometry (XRD) using 40 mA and a 40 kV current, with a monochromatic CuKα (target) radiation (λ = 0.154056 nm). XRD from (New Empyrean, Palmer naco, Netherlands) was taken at the 2θ angle range of 10–60°, and the operation condition was scan move size 0.02° and scan move time 0.05 s. The identification of functional groups in the HAP powder and the C-HAP powder was conducted with the help of the Fourier transform infrared spectroscopy (FTIR) spectrometer (WQF-510A, Changsha Tianheng Scientific Instrument Equipment Co., Ltd., Changsha, China) over a wavelength range of 4000–400 cm^−1^. In addition, the spectroscopic analysis of the PLLA powder, in comparison with the PLLA/HAP composite powder and the PLLA/C-HAP composite powder, was performed. The micromorphology of the original powder including HAP, C-HAP, PLLA, PLLA/HAP and PLLA/C-HAP was observed via scanning electron microscopy (SEM) provided by AZtecFeature, Shanghai Oxford instrument technology, Co., Ltd., Shanghai, China, and the dispersion state of the scaffold samples was also investigated to indicate the effect of CEPA. The samples were treated by spray-gold for 80 s before the SEM observation in order to make them conductive, and the impurities on the sample were carefully blown off.

The thermal stability was analyzed with a thermogravimetric (TG) analyzer from Biolin Scientific Co., Ltd., Stockholm, Sweden. The samples whose mass was about 10 mg were placed into a platinum crucible heated from the environment temperature to 650 °C at a heating rate of 20 °C/min under nitrogen condition. Considering the water contact analysis (WCA) measurement, the wettability of the scaffold samples was evaluated by an Attension Theta Lite optical tensiometer using sessile drop mode. This WCA of the scaffolds was measured at environmental temperature with a contact angle meter, and 10 μL of distilled water was slowly dropped onto the surface with a measurement time of 10 s. The tensile testing of the scaffolds was carried out on a universal mechanical testing machine running at a crosshead speed of 5 mm/min. The modulus was calculated according to the initial linear slope of the stress–strain curves. Results of three replicate samples were used to obtain the tensile strength and modulus, and then the average values and standard deviations were calculated. 

### 2.3. Bioactivity and Biocompatibility Study

The bioactivity of the PLLA/C-HAP scaffold was assessed by immersing in the simulated body fluid (SBF) solution at 37 °C for 7, 14, 21 and 28 days to observe the growth of apatite on their surfaces. The SBF solution with ion concentrations nearly equal to that of human blood plasma was prepared by dissolving NaCl, KCl, NaHCO_3_, MgCl_2_·6H_2_O, CaCl_2_, K_2_HPO_4_·3H_2_O and Na_2_SO_4_ (reagent grade, all from Chinese Academy of Sciences Beijing Chemical Reagent. Ltd., Beijing, China) into distilled water and buffered with tris hydroxymethyl aminomethane (TRIS) and hydrochloric acid (HCl) solution to pH 7.4. After immersing for different time intervals into the SBF at a concentration of 0.1 cm^2^/mL with a volume of 50 mL, the scaffolds (Φ10 mm × 5 mm) were removed, rinsed three times with deionized water and dried at 60 °C for 8 h to investigate the bioactivity.

A biocompatibility study was carried out by culturing MG-63 cells. The MG-63 cell line was provided by the American Type Culture Collection. Ltd., Manassas, VA, USA). Briefly, MG-63 cells were cultured in Dulbecco’s modified Eagle’s medium (DMEM) containing 10% fetal bovine serum (FBS) and 1% antibiotic–antimycotic solution and seeded on scaffold samples at a density of 10^5^ cells/cm^2^. After this, the cells were moved to 5% CO_2_ incubator at 37 °C. Fresh DMEM medium supplemented with 10% fetal calf serum (FCS) was added to each well to keep the cell containing the scaffold samples submerged. The plates were incubated for a scheduled time at 37 °C in a humidified atmosphere of 5% CO_2_. Finally, a fluorescence staining assay was carried out to check the cell viability. The scaffolds were extracted after 1, 3 and 5 days of culture, followed by gently washing with phosphate buffer solution (PBS). A cell adhesion test was carried out with the cultured cells/scaffold constructs. Firstly, the cells/scaffold constructs were rinsed with PBS to remove non-adherent cells for a given period. Afterward, they needed to be fixed with 2.5% paraformaldehyde and dehydrated by alcohol. Finally, the cultured cells/scaffold constructs were put into the electrothermal blowing dry box at 45 °C for 20 h. The micromorphology of the cells was characterized by SEM.

### 2.4. Statistical Analysis

Data were given as the mean ± standard deviation (SD), unless otherwise stated. Statistical analysis was performed using SPSS 19.0 software (IBM Corporation, Armonk, NY, USA), with labels * representing *p* < 0.05, ** representing *p* < 0.01 and *** representing and *p* < 0.001, respectively.

## 3. Results and Discussion

### 3.1. Surface Modification of HAP with CEPA

The modification of HAP to fabricate C-HAP powder was shown in Figure 1a. In brief, firstly HAP was dispersed in ethanol, and then CEPA was added with a high-intensity ultrasonic probe for ultrasonic oscillations, and the mixture of HAP and CEPA was of a 1:1 molar ratio. After ultrasonic treatment, magnetic stirring was carried out to disperse this C-HAP powder in ethanol. Finally, the mixed solution was filtered and dried to obtain C-HAP powder. FTIR spectra of the HAP powder and C-HAP powder are illustrated in Figure 1b. The spectra of two kinds of powder were similar, but by comparing them carefully, there were some new bonds to verify the success of grafting CEPA on the surface of HAP. In particular, the peak at 3748 cm^−1^ in these C-HAP spectra was an indication of the –OH groups belonging to CEPA. In addition, the appearance of bands for –COOH at 1558 cm^−1^ in these samwe C-HAP spectra also indicated the peaks of CEPA in the C-HAP [16]. The X-ray diffraction pattern of HAP powder and C-HAP powder was presented in Figure 1c. There was no big difference in the shift of peaks in the XRD pattern of HAP and C-HAP. However, the intensity of C-HAP peaks augmented rather than shifted compared with HAP indicated that the grafting of CEPA did not change the HAP crystal structure and the crystallinity. The schematic diagram of modifying the HAP powder was shown in Figure 1d. HAP is an ionic crystal with Ca^2+^ and (PO_4_)^3−^, and isotopic exchange studies showed that phosphonate ions, RPO_3_^2−^, could exchange with phosphate ions in HAP [17]. Therefore, in an aqueous solution, the phosphonic acid group (–PO(OH)_2_) of CEPA can produce (–PO_3_)^2−^ to form an electrovalent bond with the Ca^2+^ of HAP, which is known as Ca–O–P. The formation of interfacial bonding would be beneficial for the improvement of performance. 

### 3.2. Fabrication of PLLA/HAP and PLLA/C-HAP Composite Scaffold

**Figure 2:**
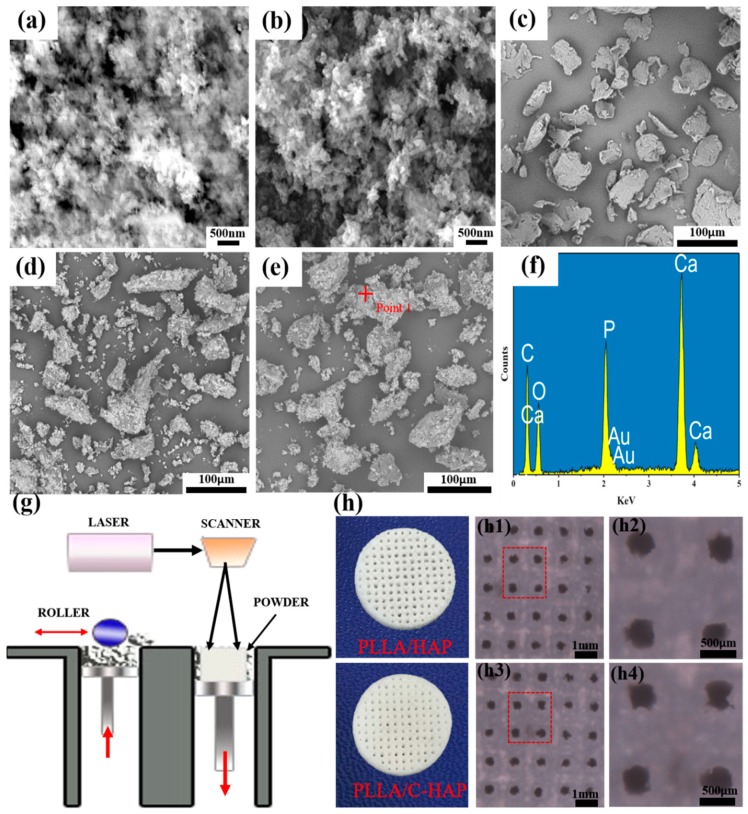
SEM morphology of (**a**) HAP nanoparticles, (**b**) C-HAP nanoparticles, (**c**) PLLA powder, (**d**) PLLA/HAP powder and (**e**) PLLA/C-HAP powder, (**f**) EDS spectra of p1 site in (**e**), (**g**) Scaffold fabricating via SLS, (**h**) Morphology of PLLA/HAP and PLLA/C-HAP scaffolds and the corresponding SEM images.

SEM morphologies of HAP, C-HAP nanoparticles, PLLA powder, PLLA/HAP and PLLA/C-HAP composite powder were shown in Figure 2a–e, respectively, and energy-dispersive X-ray spectroscopy (EDS) spectra at the p1 site was provided in Figure 2f. There was no obvious difference in the morphologies of HAP and C-HAP nanoparticles from Figure 2a,b. As was shown in Figure 2d,e, C-HAP nanoparticles were relatively more uniformly distributed in PLLA powder than were HAP nanoparticles, which might be due to the fact that the surface modification of HAP improved interfacial bonding. The scaffolds were fabricated by SLS using the powder shown in Figure 2g and the morphologies of porous PLLA/HAP and PLLA/C-HAP scaffolds are shown in Figure 2h. The PLLA/C-HAP scaffold displayed a little light yellow, while the PLLA/HAP scaffold was pure white, since the color of the CEPA was yellow. In order to promote bone regeneration, suitable structural properties and biomaterial composition are needed for the scaffold [18,19]. In particular, the interconnecting porous structure of the scaffold plays an important role in the growth of bone tissue [20,21]. In this study, the pore size, porosity, and other structural properties were simply controlled by the three-dimensional printing system. As reported in some studies, pore sizes between 200 and 400 μm were proposed as optimal for osteoconduction. Sara M et al. demonstrated that a pore size greater than 300 μm was beneficial for osteogenesis by facilitating capillary formation [22]. In order to analyze the pore size, SEM images of the PLLA/HAP and PLLA/C-HAP scaffolds were shown in Figure 2(h1–h4). It could be seen that the pore size of them was about 350 μm and was evenly distributed, which was especially beneficial for bone formation and regeneration. There was no big difference in the pore structure of PLLA/HAP and PLLA/C-HAP scaffolds, indicating that adding C-HAP had little negative influence on scaffold processing and shaping.

### 3.3. Distribution of HAP and C-HAP in the PLLA Matrix

**Figure 3:**
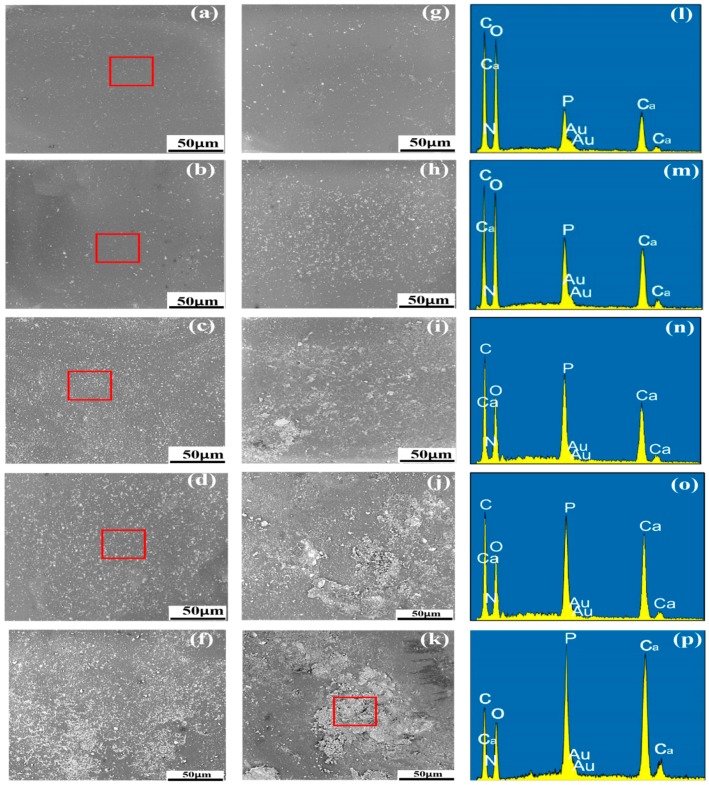
The distribution of C-HAP and HAP nanoparticles in the PLLA matrix with different filler content (**a**) 5% C-HAP, (**b**) 10% C-HAP, (**c**) 15% C-HAP, (**d**) 20% C-HAP, (**e**) 25% C-HAP, (**f**) 5% HAP, (**g**) 10% HAP, (**h**) 15% HAP, (**j**) 20% HAP, (k) 25% HAP; (**l**–**p**) EDS mapping of corresponding square area.

The dispersion of nanofiller in the polymer matrix is very important for the good mechanical performance of the scaffold. The distribution of the different content of C-HAP and HAP nanoparticles in the PLLA matrix was observed by SEM (Figure 3). Generally speaking, compared to the PLLA scaffold with HAP, there was no obvious aggregation in the PLLA scaffold with C-HAP. As for the PLLA scaffold with HAP, when the content of HAP was up to 15%, there existed aggregation in the PLLA matrix, and the aggregation became larger and more serious with the increase of HAP. As shown in Figure 3c, the Ca and P contents of HAP were obviously above that of the C and O contents, which might indicate the serious aggregation of HAP nanoparticles [23]. The aggregation of HAP was caused by a large specific surface area and high surface energy [24]. Moreover, as a hydrophilic bioceramic, HAP was incompatible with hydrophobic polymers [25,26]. On the contrary, C-HAP could be easily dispersed in the PLLA matrix, and there was only a tendency to begin to form aggregates when the content of C-HAP was up to 25%. 

The reason might be that effective interfacial bonding promoted the respective dispersion of C-HAP in the PLLA matrix on one hand, and on the other hand, modifying HAP with CEPA made C-HAP more hydrophobic than HAP, improving the compatibility for good dispersion.

### 3.4. Mechanical Properties

**Figure 4:**
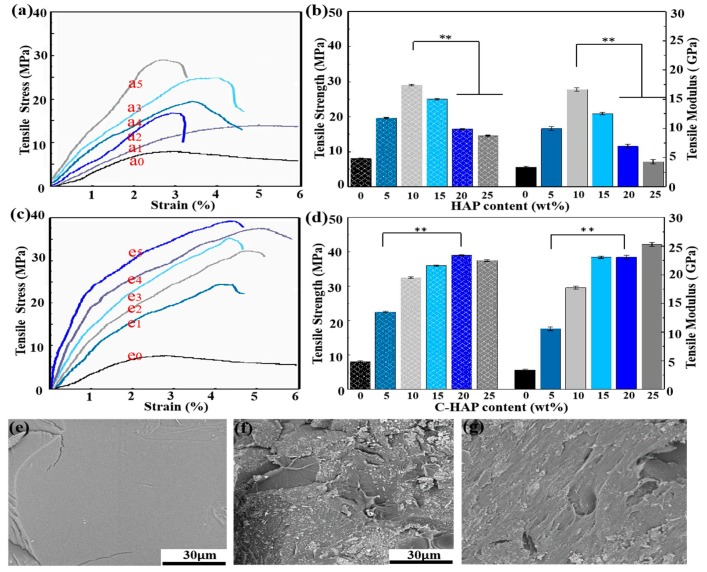
Typical tensile strain–stress curve of PLLA scaffolds with different contents of (**a**) C-HAP, (**b**) HAP, (**c**) tensile strength and (**d**) tensile modulus of the scaffolds, fracture surface of (**e**) PLLA scaffold, (**f**) PLLA/20% HAP scaffold, (**g**) PLLA/20% C-HAP scaffold.(where ** representing *p* < 0.01)

Both the mechanical strength and the modulus are important factors for scaffold application in bone tissue engineering, since scaffolds need to have enough mechanical properties to bear the weight and to maintain its dimensional integrity during cell culture [27]. The tensile properties of scaffolds were shown in Figure 4. In general, all of the PLLA scaffolds containing C-HAP showed a higher tensile strength and modulus than those of the scaffolds containing HAP with the same content. It was clear that the tensile strength increased with the increasing content of C-HAP when the content was less than 20%, and the highest tensile strength was 39.2 MPa. However, the tensile strength of the PLLA scaffolds containing HAP reached the highest (28.7 MPa) when the content of which was 10%. As reported by some studies, the tensile strength was affected by both the scaffold porosity and the material [28]. 

For the same scaffold type, the PLLA/C-HAP scaffold possessed a higher tensile strength and modulus than the PLLA/HAP scaffolds, and this was mainly due to the better dispersion states and effective interfacial bonding. Apart from improving the biological properties, the ceramic phase was added in an attempt to also improve the mechanical properties of scaffold [29,30,31]. However, aggregates would hinder the reinforcing effects contributing to uneven stress transfer [32]. As shown in SEM images of the tensile fractured surfaces, the PLLA scaffold in Figure 4e exhibited a smooth and flat surface resulting from the inherent brittle nature. However, the PLLA/20% HAP scaffold in Figure 4f showed a fairly rough morphology which indicated the plastic deformation of the scaffold. As reported by some studies, bonding strength at the interface had a great influence on mechanical properties [33,34,35]. In this study, the PLLA/20% C-HAP scaffold in Figure 4g exhibited a rough morphology with the interconnected morphology of C-HAP being pulled out from the PLLA matrix, implying a good chemical interaction. 

**Figure 5:**
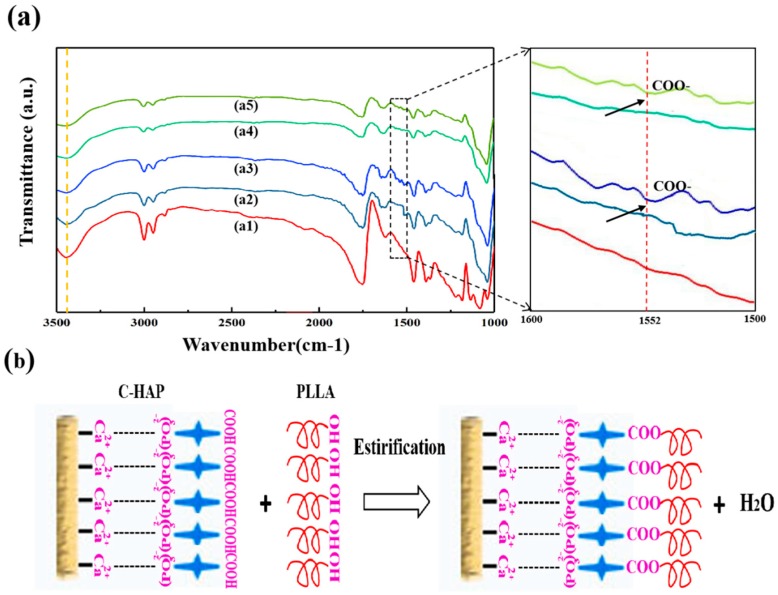
(**a**) FTIR spectra of different scaffolds (a1) PLLA scaffold, (a2) PLLA + 10% HAP scaffold, (a3) PLLA + 10% C-HAP scaffold, (a4) PLLA + 20% HAP scaffold, (a5) PLLA + 20% C-HAP scaffold, (**b**) The possible schematic of the possible interactions between C-HAP and PLLA matrix.

The existence of COO– groups on the PLLA/20% C-HAP (Figure 5a) and PLLA/10% C-HAP (Figure 5d) scaffolds was demonstrated by FTIR spectra analysis at the peak of 1552 cm^−1^. This might be ascribed to the fact that CEPA performed an esterification reaction with PLLA, attributing to strong interfacial bonding between matrix and fillers. What is more, in the spectrum of PLLA as shown in Figure 5(a1), the weak band at 3446 cm^−1^ was attributed to the stretching vibration of a terminal hydroxyl group (–OH) [36]. By comparing the FTIR spectra results of the PLLA/10% HAP and PLLA/10% C-HAP scaffolds, there was a decrease in the intensity of the hydroxyl peak at 3446 cm^−1^ in the PLLA/10% C-HAP scaffold. The same tendency was obtained that the hydroxyl peak at 3446 cm^−1^ reduced for PLLA/20% C-HAP scaffold compared to PLLA/20% HAP scaffold. 

The FTIR results indicated the esterification reaction between the hydroxyl group of PLLA and the carboxyl of CEPA occurred as reported by other studies [37,38]. The detailed process was shown in Figure 5b. Based on the above results and discussions, the mechanical properties of the scaffolds mainly depend upon the interface bonding between filler and matrix.

### 3.5. WCA, XRD and TGA Analyses

**Figure 6:**
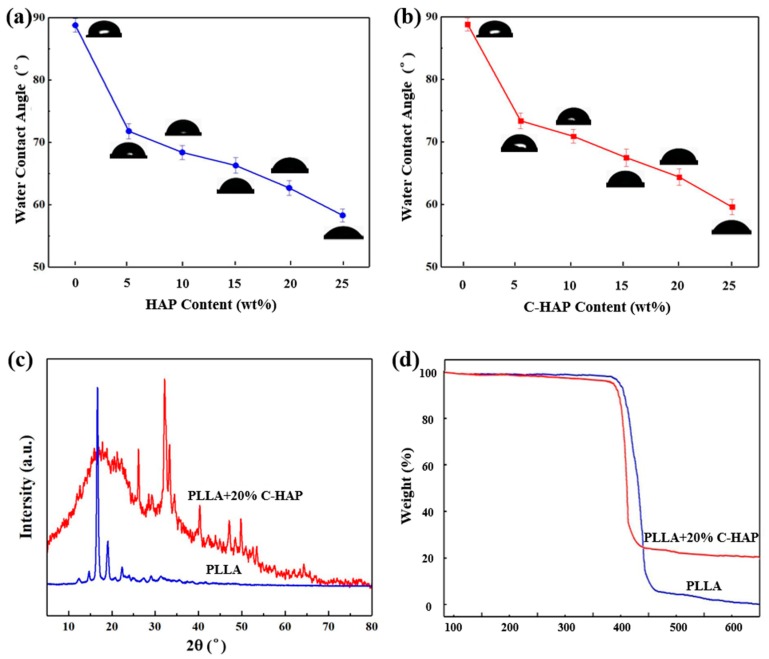
Water contact analysis (WCA) of PLLA scaffolds with different contents of (**a**) HAP, (**b**) C-HAP, (**c**) XRD pattern of PLLA/20% C-HAP and PLLA scaffolds, (**d**) TGA analysis of PLLA/20% C-HAP and PLLA scaffolds.

As we know, the wettability of material surfaces is a fundamental property for tissue engineering, and it has effects on the interactions between cells and material. According to some reports, the hydrophilic surface was better for osteoblast growth and mineral deposition than the hydrophobic surface [39], which was closely related to the bioactivity and cytocompatibility of the scaffold. As shown in Figure 6a,b, the PLLA scaffold was hydrophobic, whose WCA was about 88.7°. Compared with the PLLA scaffold, the hydrophilicity of composite scaffolds with different contents (5%–25%) of HAP or C-HAP increased nearly linearly, and the WCA of PLLA/25% HAP and the PLLA/25% C-HAP scaffold was 59.6° and 58.3°, respectively. The reason that the adding of C-HAP reduced the hydrophilicity compared with HAP might be that the esterification reaction between the (–COOH) of CEPA and the (–OH) of HAP decreased the number of hydrophilic groups [40]. The WCA of composite scaffolds were all less than 80°, which indicated that hydrophobic materials could be modified by adding hydrophilic ceramic materials. Figure 6c showed XRD patterns of the PLLA/20% C-HAP and PLLA scaffolds. The original crystalline peaks of PLLA at 2θ = 17.0°, 18.8°, 22.2° and 28.9° were assigned to the (200)/(110) and (014)/(203) crystal planes of PLLA. After adding with C-HAP particles, there appeared some new peaks at 2θ = 26.1°, 32.0°, 33.2° and 40.2°, which were belonging to HAP, and some displacements indicated the change of crystal structure after forming interfacial bonding [41]. The wide diffraction peak (10°< 2θ < 20°) of the composite could be attributed to amorphous PLLA [42,43]. Thermal analysis of PLLA and PLLA/25% C-HAP scaffolds was performed to identify thermal stability. 

The degradation onset temperature and degradation temperature were decreased comparing the degradation properties of the PLLA/25% C-HAP scaffold with the PLLA scaffold. The electrostatic attraction between Ca^2+^ ions and (PO_3_)^2−^ might be responsible for the reduced thermal stability of the PLLA/25% HAP scaffold, compared with the PLLA scaffold [44]. From the TGA curve, we could see that in nitrogen atmosphere there was nearly no mass loss before 200 °C, which was no indication for the material to decompose. Therefore, it could be concluded that the scaffolds had good thermal stability. Some studies revealed that the degradation of the polymer matrix occurred in a major weight loss process in the range of 300–400 °C. The comparison between the residual weight of the PLLA/25% C-HAP and PLLA scaffolds suggested that the amount of C-HAP ceramic presented in the PLLA matrix was around 20%.

### 3.6. Bioactivity and Cytocompatibility

**Figure 7:**
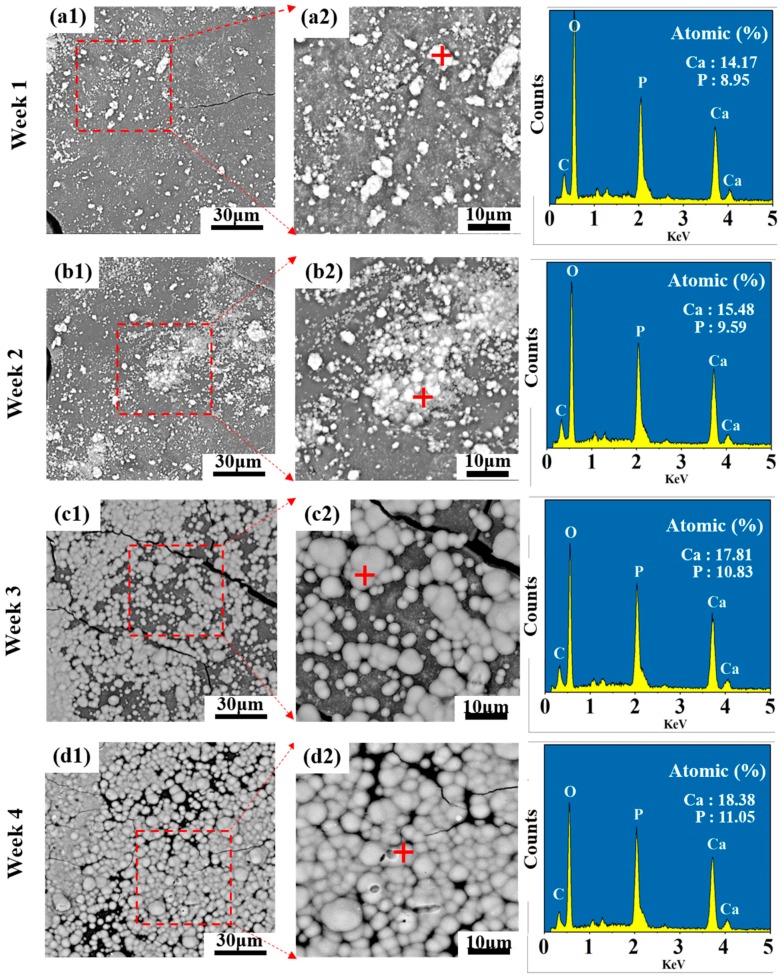
Bioactivity of the PLLA/20% C-HAP scaffold after SBF immersion for (**a1**) 7 days, (**b1**) 14 days, (**c1**) 21 days and (**d1**) 28 days. (**a2**), (**b2**), (**c2**), and (**b2**) are the corresponding enlarged image corresponds to the rectangular area of the graph.

Apatite layer formation of the PLLA/20% C-HAP scaffold was investigated by SEM after immersion for a periodic time. It could be seen from Figure 7 that the PLLA/20% C-HAP scaffold owned a good bioactivity to induce apatite precipitation. The apatite layer became larger and thicker as time went on from (a1) to (d1) in Figure 7. The increased Ca P atomic content in the EDS spectra of the corresponding square area also revealed the increase of the apatite layer, and the Ca/P ratio of each scaffold with increasing immersion time was 1.58, 1.61, 1.64 and 1.66, respectively. From some studies we knew that biodegradable PLLA polymer was of no bioactivity, thus we speculated the good bioactivity of scaffolds was owing to C-HAP. Some reports revealed that the bioactivity of HAP was attributed to the dissolution of the Ca^2+^ and PO_4_^3−^ ion acting as deposition sites of apatite to precipitate crystals in the SBF solution [45,46]. Thus, the accumulation of calcium ions created a positively-charged surface attracting negative phosphate ions from the SBF solution. What is more, the PLLA/20% C-HAP scaffold contained a phosphonic acid group (–PO(OH)_2_), which had a strong affinity for Ca^2+^ in the SBF solution to induce apatite nucleation.

**Figure 8:**
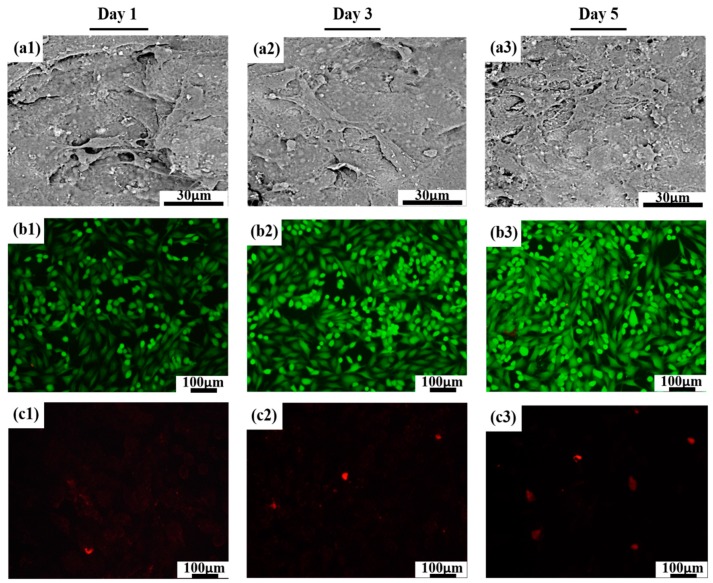
Adhesion morphology of MG-63 cells cultured on the PLLA/20% C-HAP scaffold for (**a1**) 1 day, (**a2**) 3 days, (**a3**) 5 days; Fluorescence staining of live MG-63 cells after culturing on the PLLA/20% HAP scaffold for (**b1**) 1 day, (**b2**) 3 days, (**b3**) 5 days; Fluorescence staining of dead MG-63 cells after culturing on the PLLA/20% C-HAP scaffold for (**c1**) 1 day, (**c2**) 3 days, (**c3**) 5 days.

SEM was used to illustrate the morphology of MG-63 cells attached to the surface in Figure 8a1–a3. In general, MG-63 cells exhibited an extended and well-spread morphology and intimately attached to the surface of the PLLA/20% C-HAP scaffold. Additionally, as time went on, the numbers of cells were denser, and cells maintained a more close physical contact with one another. In addition, it could be seen that MG-63 cells produced some secretion on the scaffold. Besides, immunofluorescence was used to analyze the viability of MG-63 cells. The live MG-63 cells were stained to green and the dead MG-63 cells were stained to red. From the results, we knew that the number of cells gradually increased with the culture time increasing, which indicated the scaffold was nontoxic and beneficial for cell growth [47]. Some reports showed that interconnected and porous structures of the scaffold had the potential to support cell proliferation and growth [48,49]. The PLLA scaffold was a lack of cytocompatibility, thus the reason might be that HAP was a kind of bioactive ceramic rich in Ca^2+^ and (PO_4_)^3−^ to enhance the binding of cell and scaffold [50]. In our experiment, the adhesion, as well as proliferation of MG-63 cells, was fairly good by adding 20% C-HAP into the PLLA matrix, but in the study of Smieszek et al. [51], porous PLLA scaffolds with 10 wt % HAP could significantly improve the adhesion and proliferation of adipose-derived stromal cells. Our results differ, not only because of the methods used to prepare bone scaffold, but also because MG-63 cells have special cellular physiological characteristics. Therefore, the experiments indicated that the culture microenvironment modulated by incorporating C-HAP into the PLLA matrix was beneficial for MG-63 cell adhesion, growth and proliferation.

## 4. Conclusions

In this study, HAP was modified by a CEPA phosphonic acid coupling agent acting as a cross-linking bridge to improve the weak interfacial interaction between PLLA and HAP, and composite scaffolds were fabricated by SLS for their applyication to bone tissue engineering. After modification, HAP achieved an even dispersion in the PLLA matrix, and the interconnected morphology of C-HAP pulled out from the PLLA matrix suggested improved interfacial bonding. As a result, the tensile strength and modulus of the scaffold with 20% C-HAP reached 39.5 MPa and 26.4 GPa comparing to that PLLA scaffold of 8.1 MPa and 3.4 GPa, respectively. Furthermore, the PLLA/20% C-HAP scaffold possessed good bioactivity in the deposition of the apatite layer in the SBF solution and good cytocompatibility in the growth of MG-63 cells. 

## Figures and Tables

**Table 1:** Material compositions of the composite powder.

Filler Formulations	Material Mass (g)			
	PLLA/HAP Composite		PLLA/C-HAP Composite	
	PLLA	HAP	PLLA	C-HAP
0%	10	0	10	0
5%	9.5	0.5	9.5	0.5
10%	9	1	9	1
15%	8.5	1.5	8.5	1.5
20%	8	2	8	2
25%	7.5	2.5	7.5	2.5
